# PangenomeNet: a pan-genome-based network reveals functional modules on antimicrobial resistome for *Escherichia coli* strains

**DOI:** 10.1186/s12859-021-04459-z

**Published:** 2021-11-10

**Authors:** Hsuan-Lin Her, Po-Ting Lin, Yu-Wei Wu

**Affiliations:** 1 Bioinformatics and Systems Biology Program, University of California San Diego, La Jolla, CA 92093 USA; 2 Department of Mechanical Engineering, National Taiwan University of Science and Technology, No.43, Keelung Rd., Sec.4, Da’an Dist., Taipei City, 10609 Taiwan; 3 Center for Cyber-Physical System Innovation, National Taiwan University of Science and Technology, Taipei, 10609 Taiwan; 4 Graduate Institute of Biomedical Informatics, College of Medical Science and Technology, Taipei Medical University, 250, Wuxing St., Sinyi District, Taipei, 11031 Taiwan; 5 Clinical Big Data Research Center, Taipei Medical University Hospital, Taipei, 11031 Taiwan

**Keywords:** PangenomeNet, Pan-genome, *Escherichia coli*, Functional network, Antimicrobial resistance

## Abstract

**Background:**

Discerning genes crucial to antimicrobial resistance (AMR) mechanisms is becoming more and more important to accurately and swiftly identify AMR pathogenic strains. Pangenome-wide association studies (e.g. Scoary) identified numerous putative AMR genes. However, only a tiny proportion of the putative resistance genes are annotated by AMR databases or Gene Ontology. In addition, many putative resistance genes are of unknown function (termed hypothetical proteins). An annotation tool is crucially needed in order to reveal the functional organization of the resistome and expand our knowledge of the AMR gene repertoire.

**Results:**

We developed an approach (PangenomeNet) for building co-functional networks from pan-genomes to infer functions for hypothetical genes. Using *Escherichia coli* as an example, we demonstrated that it is possible to build co-functional network from its pan-genome using co-inheritance, domain-sharing, and protein–protein-interaction information. The investigation of the network revealed that it fits the characteristics of biological networks and can be used for functional inferences. The subgraph consisting of putative meropenem resistance genes consists of clusters of stress response genes and resistance gene acquisition pathways. Resistome subgraphs also demonstrate drug-specific AMR genes such as beta-lactamase, as well as functional roles shared among multiple classes of drugs, mostly in the stress-related pathways.

**Conclusions:**

By demonstrating the idea of pan-genome-based co-functional network on the *E. coli* species, we showed that the network can infer functional roles of the genes, including those without functional annotations, and provides holistic views on the putative antimicrobial resistomes. We hope that the pan-genome network idea can help formulate hypothesis for targeted experimental works.

**Supplementary Information:**

The online version contains supplementary material available at 10.1186/s12859-021-04459-z.

## Introduction

Antibiotic resistance is an emerging worldwide problem. Due to the misuse of antibiotics, emergence of highly resistant pathogens has again transformed the once conquered infectious diseases lethal. To control those resistant pathogens, new drugs are needed. However, new antimicrobial agents approved by the Food and Drug Administrative is declining since the 1990s [1], hinting that we are running out of drugs against AMR pathogens very quickly, as exemplified by the hospital superbug, methicillin-resistant *Staphylococcus aureus* (MRSA) [2].

To generate new strategies against resistance, we must know more about resistance mechanisms—how do those mobile elements and mutations change the dynamics of microbes? Are there new resistance genes within the pathogens that mandate therapeutic targeting? How quickly and how often do pathogens acquire AMR genes? It has been hypothesized that antibiotic resistance trades off against fitness in the absence of antibiotics [3, 4], in which adaptive genes amplify against antimicrobial agents and are accompanied by compensatory mutations to tackle the fitness losses [5]. This indicates that the acquisition of resistance genes exposes new vulnerability in pathogens that may allow new strategies against resistance strains.

Networks are becoming powerful tools in functional genomics [6], hypothesis generation in disease research [7], and gene essentiality prediction [8–10]. For example, recent studies have identified novel genes and potential drug target molecules from network analysis [11]. Existing networks, however, suffer from one major drawback: many genes without known functions are missed in the species-level networks; as a result the networks are biased toward the core genome [12–14]. In other words, genes with unknown functions (often annotated as hypothetical genes) are often excluded from the analysis and hence cannot be inferred for functional purposes. However, many resistance determinants are acquired from horizontal gene transfer and do not belong to the core genome. Furthermore, existing network-based approaches usually focus on one or several strains instead of utilizing a more comprehensive collection of strain-level variability, resulting in potential biases toward certain strains.

In order to identify hypothetical genes that may be related to AMR activities from a more comprehensive collection of pathogenic strains, we incorporated the idea of pan-genome, which is defined as the collectively shared genes of all strains of a certain bacterium, and used it to build a co-functional network. This allowed us to include all available genetic elements, including putative genes with unknown functions (hypothetical genes), into the network for analysis purpose. Our network (PangenomeNet) was constructed based on information from three different data types: co-inheritance information, protein domain sharing, and protein–protein interactions. Each type of the data has the potential to complement other data types (i.e. providing gene relationships that can only be seen within one of the data types) and provides a functional organization of the resistome. By building the pan-genome for the common pathogen *Escherichia coli* and using the pan-genome to build a co-functional network, we show that we are able to predict the functions for putative hypothetical genes and demonstrate their functional links with antibiotic resistances.

## Results

### The *Escherichia coli* pan-genome

**Fig. 1:**
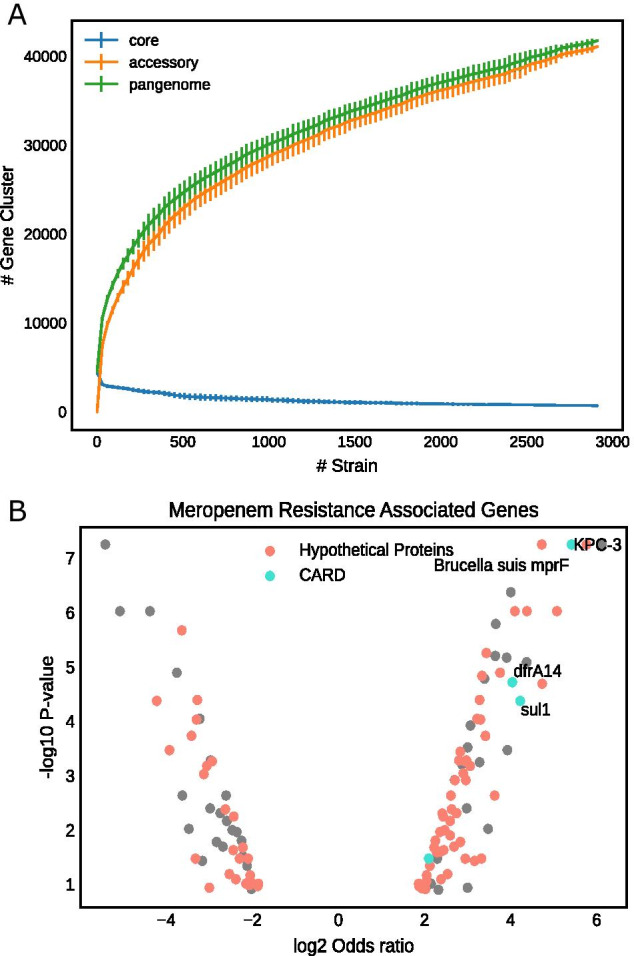
Pan-genome statistics and meropenem-resistance genes. **A** Growth curves of the core, accessory and pangenome; **B** Scoary-predicted meropenem-resistance associated genes

We built a pan-genome for the *E. coli* species in order to probe thousands of strains at the same time. As shown in Table 1, the constructed *E. coli* pan-genome, which was built from 2931 *E. coli* genomes (list of genomes and their genomic properties can be found in Additional file 2: Table S1), consists of 41,822 gene clusters, among which 3056 belong to the core-genome and 38,766 belong to the accessory genome (Table 1).   After annotating gene clusters for their functional roles (Additional file 3: Table S2), we identified that 48.86% of the gene clusters are designated as hypothetical proteins. The growth curves of core-, accessory-, and pan-genomes were plotted to check whether our “extended core (99% identity)” definition [15] is suitable for ongoing pan-genome analysis. By fitting to the power law distribution we identified that the *E.coli* pan-genome is an open pan-genome (alpha = 0.21) (Fig. 1A, Additional file 1: Fig. S1) [16], indicating the highly diverse genetic variability among different *E. coli* strains.

**Table 1:** *Escherichia coli* pan-genome statistics

Genome number	Pangenome size	Core size	Accessory size	Hypothetical (%)
2931	41,822	3056	38,766	20,436 (48.86%)

To probe the connection between gene clusters and the AMR phenotypes, we used pangenome-wide association tool, Scoary [17], to identify gene clusters that are significantly correlated with antimicrobial resistance phenotype. Using meropenem resistance as an example, Scoary identified 683 putative carbapenem-resistance associated genes, with KPC Carbapenemase as the strongest resistance determinant (Fig. 1B). Only 30% of the putative resistance genes have GO annotation, among which 1.2% can be annotated by Comprehensive Antibiotic Resistant Database (CARD) [18] and 3% are detected by Resfam [19]. There are many hypothetical genes that are significantly associated with meropenem resistance (Fig. 1B). Similar observations were also made on other antibiotics, in which many hypothetical genes are strongly associated with drug resistances (Additional file 1: Fig. S2, Additional file 4: Table S3). GO enrichment analysis demonstrated enrichment in aromatic compound catabolism (GO:0019439) and C4-dicarbozylase transport (GO:0014730) (Additional file 1: Fig. S3, Additional file 5: Table S4). Since only a small set of AMR genes can be annotated by existing databases, we reasoned that a more comprehensive functional view on meropenem resistance requires an alternative approach.

### The PangenomeNet

In order to identity the functional roles of the hypothetical genes that are potentially related to antimicrobial resistance activities (termed hypothetical AMR genes hereafter) in *E.coli*, we built and integrated a co-functional network using the pan-genome based on three different types of information: co-inheritance [20], domain sharing [21], and protein–protein interaction relationship [22] (See Methods for details). As the complexity of network construction grows quadratically with the number of gene clusters, we reduced computational load by selecting 2052 accessory genes that were associated with resisting at least one drug (detected by Scoary) along with 1001 sampled core genes to construct the network.

Even though the amount of hypothetical genes of the co-inheritance networks (including the RefseqNet and the EskapeNet) and DomainNet are significant (approximately 20–28%), the two networks are fragmented (as indicated by the number of components). In order to improve the connectivity of the network, the STRING network (STRINGNet) [22] was integrated with the two co-inheritance networks. As shown in Table 2, the coverage of the integrated network (termed PangenomNet) reaches 2284 out of 3053 (74%) gene clusters, among which 1001 (43.8%) belong to the core genome and 1283 (56.2%) belong to the accessory genome.

**Table 2:** Properties of individual networks and the composite PangenomeNet

No. nodes	Nodes^a^	Components	Core	Accessory	Hypothetical
Total^b^	3053	–	1001	2052	1122
RefseqNet	1834	5	1001	833	225
EskapeNet	1960	9	997	963	321
StringNet	1118	1	982	136	105
DomainNet	2131	35	994	1137	319
**PangenomeNet**	**2284**	**12**	**1001**	**1283**	**424**
EcoliNet	1095	5	964	131	0
Mentha	782	64	683	99	0

Bold indicates the properties of PangenomeNet built by this work

^a^Indicates gene clusters constructed from the pan-genome

^b^Indicates the 1001 core genes along with the 2052 accessory genes associated with at least one drug (selected by Scoary)

**Fig. 2:**
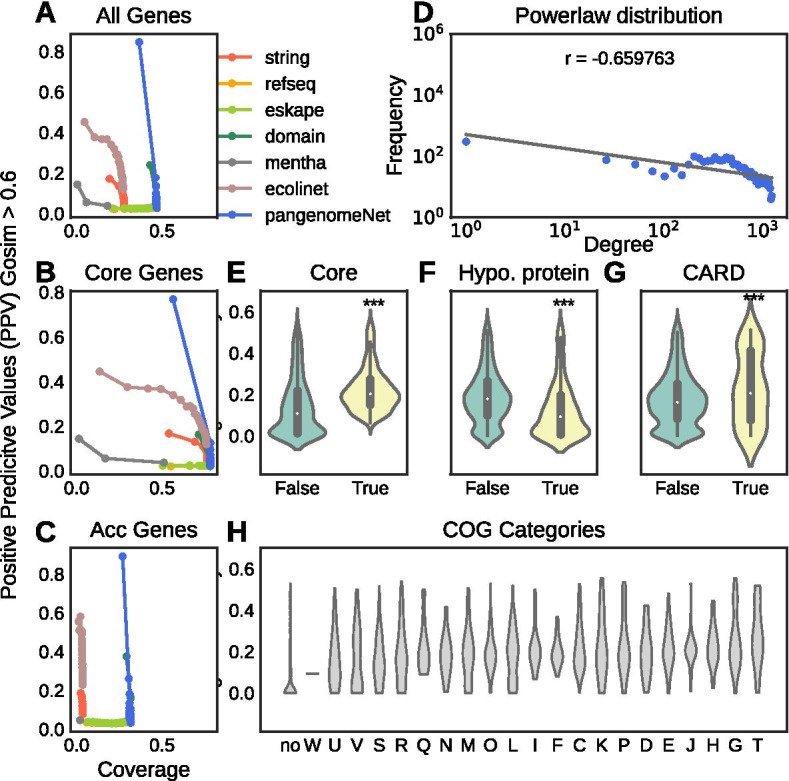
The network statistics and properties of PangenomeNet. Positive-predictive values (PPV) and coverage tradeoff curve for **A** the entire pangenome, **B** core genes, and **C** accessory genes; **D** Power-law distribution of the Pan-genomeNet; **E–H** Degree centrality score for different sets of genes

GO term semantic similarity score (GOSim) [23, 24] was calculated to evaluate the accuracy of the PangenomeNet for co-functional inferences. The score distributions of all individual networks are positively associated with log-likelihood scores (LLS; GOSim > 0.6 is labelled as true interaction), indicating that all four networks contain information in determining the GO similarity (Additional file 1: Fig. S5). Since each the four networks contributes to unique nodes and edges (Additional file 1: Fig. S6), we reasoned that all components are indispensable to a comprehensive view on the pan-genome. As shown in Fig. 2A–C, the integrated network (PangenomeNet) is more comprehensive than individual networks. Comparing to EcoliNet [25], which is a co-functional network built on 4146 protein-coding genes from *E. coli*, and Mentha, an expert-curated protein–protein interaction database [26], the PangenomeNet has better performance for covering both core and accessory genes. We note that Mentha and STRING networks had very poor positive predictive value (PPV)-coverage tradeoff for accessory genes, showing their biases towards the core genome.

The scale-free property, defined by the power law distribution (the probability a node has *k* links $P(k) {k}^{-t}$ with *t* as the degree component) [6], was also checked since most biological networks are scale-free. As shown in Fig. 2D (the power law distribution for all networks are available in Additional file 1: Fig. S7), the PangenomeNet fits the characteristics of biological networks and can be used for functional inferences.

### Network centrality scores

Since genes that are critical to the survival of bacteria tend to be centralized in the gene or protein networks [27], the centrality score of several gene cluster groups were examined. Not surprisingly, we identified that core genes receive significantly higher centrality scores compared to accessory genes (Fig. 2E, Kolmogorov–Smirnov *p* value 2e−85), and that hypothetical genes have lower centrality scores (Fig. 2F, Kolmogorov–Smirnov p-value 7e-232). COG categories that are essential to bacterial survival (ECFHKMLGIJ) also receive significantly higher centrality (Fig. 2H, Kolmogorov–Smirnov *p* value 1e−147) [28]. These results again suggest that the PangenomeNet is consistent with the looks of biological networks.

The centrality scores of AMR genes were also checked. As shown in Fig. 2G, The AMR genes annotated by the CARD database have higher centrality than ordinary accessory genes, but lower than core genes (Kolmogorov–Smirnov *p* value 0.0003). The results suggests that the AMR may be located at the periphery of core genes, indicating their close connection to normal physiology [29].

### PangenomeNet reveals functional organization of the meropenem resistome

**Fig. 3:**
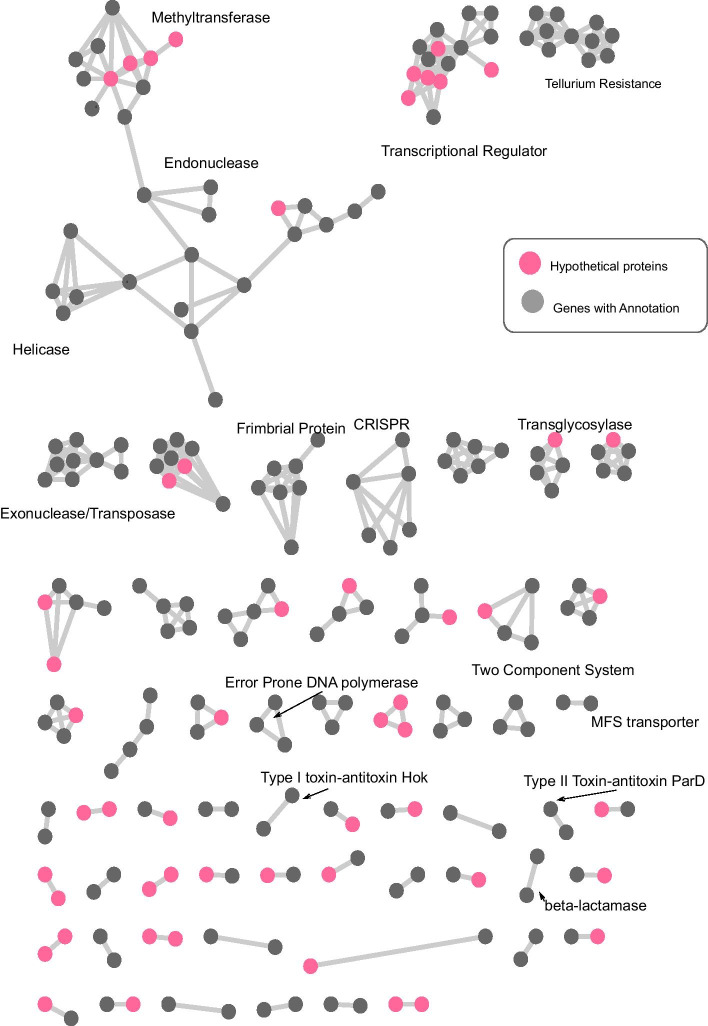
Meropenem resistance gene subnetworks. Grey nodes indicate genes with annotations while pink nodes indicate hypothetical proteins. Functional overviews of the sub-components are indicated next to the components

We extracted a subnetwork with 683 putative resistance genes (see Methods; network details provided in Additional file 6, Table S5 and Additional file 7, Table S6) for a complete functional view of meropenem resistome. With predicted GOsim > 0.6, our PangenomeNet is able to cover interactions among 224 (32.7%) of them. In contrast to common resistance gene annotation tools, including CARD [18] and Resfam [19], which annotate 1% and 3% of all putative resistance genes, our network has the advantage in discovering novel resistance modules. The subnetwork consists of a large connected component along with several small components (Fig. 3; detailed network with node annotations is also available on Network data exchange (NDX); see Availability of data and materials for accession). The largest component consists of several densely connected communities, indicating resistance acquisition is functionally modularized.

### The Meropenem resistome contains a cluster of new drug target, transglycosylase

Our network identified a separate component that is composed of three transglycosylases**,** one conjugal transfer protein, and one hypothetical protein, as shown in Fig. 3. The prevalence of transglycoylases in the resistome also suggests the importance of this gene family. Interestingly, recent genetic screens show transglycosylase disruption increases susceptibility to beta-lactams in *Pseudomonas aeruginosa*. Bulgecin A, a small molecule transglycosylase inhibitor, can restore the efficacy of meropenem [30]. Meropenem, a type of beta-lactam antibiotic, targets penicillin binding protein, which usually has dual transpeptidase and transglycosylase activity in cell wall formation [31]. Upon cell wall perturbation, the transglycosylase cleaves the accumulated product, producing metabolites that are capable of inducing beta-lactamase resistance genes [32] and offering survival advantage upon meropenem treatment. The connections between the hypothetical proteins with the transglycosylases indicate their potential roles in cell wall formation, and may be novel targets to combat meropenem resistances.

### The Meropenem resistome contains clusters related to signal transduction pathways to stress response and gene acquisition

**Fig. 4:**
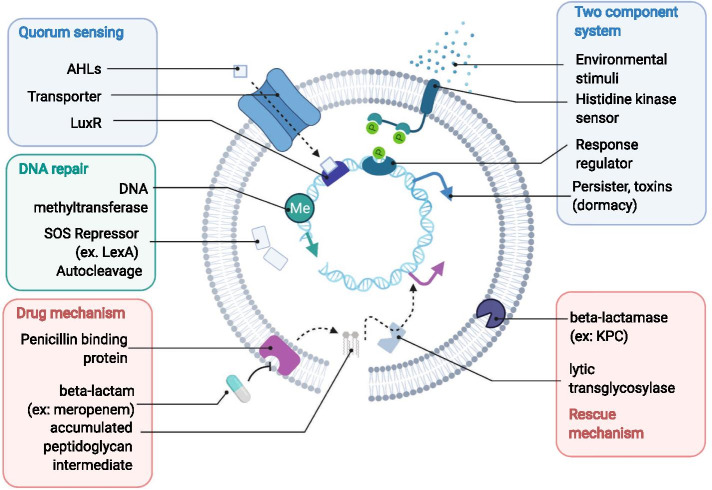
Inferred mechanisms for meropenem resistance acquisition revealed by this study

Several subnetworks consist of known proteins that elicit downstream transcriptional response for antibiotic stress adaptation (Fig. 3), including two component systems, Quorum sensing Luxl–LuxR [33], toxin–antitoxin systems (type II: HigA, ParD, HicB; type I: Hok) [34], Tellurium-resistant genes [35–39] and Willebrand factor type A (vWA) [40, 41]. These signal transduction pathways regulate cell density, virulence and dormancy [33, 34, 42, 43]. The rapid gene expression changes allow “phenotypic resistance”, that is, resistance without genetic alterations. For example, dormant cells are more tolerant to antibiotics due to lower target activities or drug uptakes [42, 43]. Such mechanisms were found to be the first line defense against antibiotics before acquiring resistance genes that removes the drug from the cells [42, 43]. We summarized the mechanisms from existing literature of how these genes lead to resistance in Fig. 4.

Several pathways related to resistance gene acquisition were also identified in the network. First, Error prone DNA polymerase V may increase mutation rate and thus allows better adaptability to stress [44]. Second, DNA methylation has been shown to enhance viability under antibiotic treatment. Absence of DNA methyltransferase impairs methyl-dependent mismatch repair, leaving the bacteria overwhelmed with deleterious DNA break [45]. Epigenetic modification is also crucial for survival under sub-inhibitory concentration, enabling flexible phenotypic resistance [46]. Interestingly, a group of DNA methyltransferase connect tightly with a group of hypothetical proteins (DUF 4942 and two other hypothetical protein), indicating that these hypothetical proteins may be a novel group of methyltransferase, or a system working in conjunction with the DNA repair process. Last but not least, large number of genes participate in horizontal-gene-transfer (HGT) mechanisms, including phage, plasmid and integron proteins that may allow genetic acquisition of resistances [47].

### Pan-resistome reveal drug-class-specific and general resistant gene modules

**Fig. 5:**
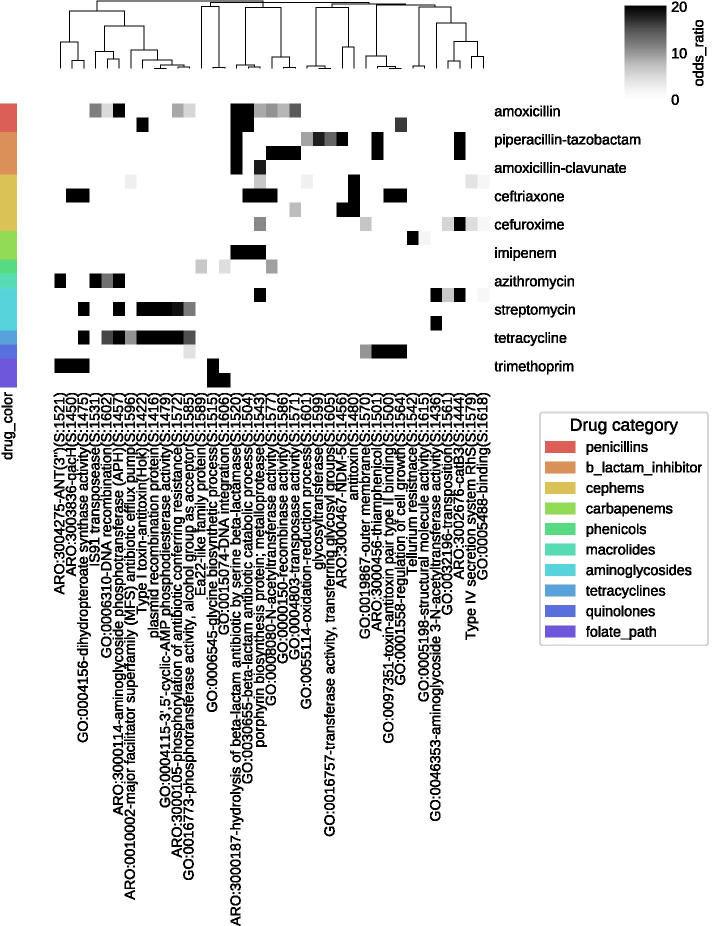
Clustering of shared terms in the pan-resistome ontology

Many of the aforementioned resistance modules such as the signal transduction and mutational related pathways are not specific to Meropenem. Motivated by this finding, we aimed to compare the resistomes detected by Scoary for all antibiotics. We extracted all 2052 putative resistance genes that are associated with any of the antibiotic drugs and constructed a subnetwork termed pan-resistome subnetwork. Similar to the meropenem resistance network, the pan-resistome network is highly modularized. To quantitatively extract functional modules, we ran data-driven ontology (DDOT) [48] to organize the network into an ontology and aligned the terms to Gene Ontology [49] and Antibiotic Resistance Ontology [48] (Additional file 8, Table S7). To identify drug-specific gene modules, for each term in the tree, we ran Fisher’s exact test to find terms that contain genes significantly associated with a specific drug. As shown in Fig. 5, we found several drug-specific terms. For example, term ARO:3000187-hydrolysis of beta-lactam antibiotic by serine beta-lactamase (S:1520) and GO:0030655-beta-lactam antibiotic catabolic process (S:1504) are associated with multiple beta-lactam drugs (cephem, penicillin and carbapenems). Genes associated with this term are annotated as various families of beta-lactamases: SHV, CTXM and TEM1.

Specific classes of beta-lactamases are associated with subtypes of beta-lactams. For example, ARO:3000467-NDM-5 (S:1456) and glycosyltransferase are only associated with cephems among the probed drugs, indicating the potential roles of these two enzymes on cephem resistances. Another example of drug specific term is GO:0006545-glycine biosynthetic process (S:1515) with enrichment in drugs trimethoprim and trimethoprim-sulfamethoxazole. Trimethoprim, a folate synthesis inhibitor, inhibits dihydrofolate reductase (DHFR) and impairs downstream glycine and methionine synthesis, hampering cell growth [50]. Genes associated with these terms are all transposon-encoded DHFR dfrA8 and drfA12.

In addition to the drug-specific resistome, we also identified many GO terms shared among drugs. For example, toxin–antitoxin system is associated with multiple drug class, including 'GO:0097351-toxin–antitoxin pair type II binding (S:1500)', 'antitoxin (S:1480)', and 'Type I toxin–antitoxin (Hok) (S:1422). None of these are annotated by CARD or Resfam. In addition, two genes belonging to Type I toxin–antitoxin Hok do not have GO annotation. This example shows that our network can complement current gene annotation databases and allow a functional overview of the resistome by grouping genes with related functions together.

## Discussion

Here we built a network on the pan-genomic scale to investigate the biology of hypothetical genes and their roles of antibiotic resistance. In our network, we found many functional modules that is related to resistance, either to a specific class of drug or serve as a general response to stress. The latter one, however, is less well-known and poorly documented in current annotation databases. Meanwhile, experiments in literature report strains with those systems display phenotypic resistance, thereby allowing more time for these pathogens to acquire genetic resistance. For example, two component systems, quorum sensing pathways and toxin–antitoxin systems utilize different signal transduction pathways to induce dormancy and virulence gene modules (Fig. 4) [33, 34, 51]. Organic or heavy metal solvent tolerance was found to be associated with multidrug resistance, possibly due to shared mechanism against drug and solvent [35, 36, 38, 39]. Deletion of Tellurium-resistant operons has been reported to reduce resistance to cell-wall-targeting drugs in *Listeria* [37]. Error prone DNA polymerase V is required for persister cells heritable resistance [44]. Unlike the traditional shield-and-weapon view of AMR genes versus antibiotics [29], our findings and literature both suggest there may be more genes related to the antimicrobial resistance activities than well-documented ones.

Since the foundation of our network is based on pan-genome, the first step for building the PangenomeNet would be the construction of the bacterial pan-genome. The most important step for building the pan-genome is clustering the genes at certain amino acid identity threshold, in which we chose 70% as the clustering identity cutoff. There are two reasons choosing 70% as our identity threshold. Firstly, we annotated Gene Ontology (GO) terms for the genes using Interproscan v5.47–82 [52] and checked the proportion of gene clusters with consistent GO terms. As shown in Additional file 9: Table S8, we found that gene clusters ranging from 95 to 70% amino acid identity were all highly consistent in their GO term functionality annotations, indicating that identity as low as 70% may also be used for pan-genome construction purpose. The second reason would be that even though our approach is very useful in inferring gene functionalities for hypothetical proteins, the time required for running our algorithm grows quadratically with the number of gene clusters. We therefore decided to choose 70% as our clustering identity threshold for both the reduced number of gene clusters and the highly-consistent GO term annotations.

Our network reproduces characters of biological networks–scale free, and has better coverage in both core and accessory components compared to STRING networks [22] by species. It captures the underlying biology of core and accessory genes, demonstrating high connections in core genes, indicating its central physiologic role. The locations of known resistance genes on the periphery of core components indicate their tight connection to modifying the physiology to generate resistance. We note that EcoliNet [25], which consists of 4099 protein coding genes that covers 99% of a single *E. coli* genome, was also constructed using similar methods.^.^ Our pan-genome-based network, however, is far more comprehensive than EcoliNet: the pan-genome composes of 41,822 gene clusters from 2931 strains. In other words, the pan-genome network can be used to describe the functional roles of the *E. coli* species in terms of thousands of strains. 

The investigation of the network connectivity reveals that the PangenomeNet, which was generated from the integration of four networks, is more comprehensive than any individual network, suggesting that utilizing different information helps in connecting the dots. The evaluation of network quality using GO term similarity scores also indicates that the integrated PangenomeNet is much more comprehensive than other networks. Other networks have high biases towards the core genome that mainly contains most of the well-studied genes. Even though our integrated network, PangenomeNet, is also somewhat biased, in which the coverage of the accessory genome is lower than the core genome, the bias is less significant than other networks. We hypothesize that such bias is an inevitable result from training on GO terms, as Gene Ontology itself is also biased. In our pan-genome, 78% of core gene clusters were annotated by Gene Ontology, while only 31% of the accessory gene clusters were annotated. The cumulative distribution of GOsim scores [23] for accessory-accessory gene pairs was also significantly lower (Additional file 1: Fig. S4).

Despite the efforts and contributions that we made in this work, there are still limitations. The first limitation of this method is that the running time grows quadratically as the size of pan-genome grows, as we are investigating all possible interactions between gene pairs, and thus will take a long time on pan-genome studies with a lot of genes or gene clusters. In this study, we demonstrated computing only on the genes of interest—the Scoary-detect resistance genes, in order to save computational resources. However, doing so omits the possibility of annotating hypothetical genes that are able to form connections with resistance genes but are not in the selected group (i.e. not detected by Scoary). Secondly, the bias toward the core genome lead to the loss of connections between accessory genes, resulting in an incomplete annotation of the whole resistome. Despite these biases, we nevertheless emphasize that the successful inference of potential antimicrobial resistance genes from the PangenomeNet shows that it is possible to probe putative AMR genes from thousands of microbial strains instead of just focused on one or a few strains. Last but not least, to thoroughly understand the mechanism of which these putative AMR genes lead to resistance, more experimental works are required.

In the future we plan to extend this work toward three directions. Firstly, we aim to develop a systematic annotation pipeline to annotate hypothetical proteins given a network, be it PangenomeNet or other networks built by other groups. This could greatly improve our annotation efficiency on any network. Secondly, we also seek to incorporate point mutation, insertion, and deletion information into the PangenomeNet in order to better utilize information on the nucleotide level. Finally, we also wish to develop an experimental platform to show the correlation between the hits and the acquisition of resistance. We hope this network can serve as a starting point of systems biology on species-level antimicrobial resistance activities and a guide on experimental biology and pharmacologic development.

## Methods

### Construction of the pan-genome

A pan-genome is defined as all possible genes that can occur within a single strain within a study group [53, 54]. As many antibiotic resistance genes disseminate horizontally, we reasoned the pan-genome includes both the core genes (genes exist in all *E. coli* strains) and the accessory genes (genes specific to a few, but not all, strains) to each strain, can best cover all possible genes conferring resistance. The construction steps are as follow: All *E. coli* genomic sequences (.fna) with resistant phenotype annotation, “Good” genome quality and from a human host were downloaded from PATRIC database website [55, 56] on February 2021. Genomes annotated as “plasmid” or contained less than 4826 genes (which is 60% of median gene number among all downloaded *E. coli* genomes) were removed from this study. The removed genomes had an average of 1274 CDS inferred. Upon checking the genome quality using checkM [57] we identified that the genome completeness of the excluded genomes were much lower than included genomes, as shown in Additional file 1: Fig. S8. In the end, a total of 2931 genomes were included in the construction of the pan-genome. The complete genome list and statistics are provided in Additional file 2: Table S1.

Protein coding sequences were predicted from all genomes using Prodigal v2.6.3 [58]. Pan-genome was then constructed by grouping genes predicted from every genome and then clustering the genes using CD-HIT v4.6 [59, 60] with amino acid identity 70%. We adapted the definition of extended core as our core genome, which corresponds to the presence in 99% of the genomes [15]. The representing genes of the clusters grouped by CD-HIT were annotated using DIAMOND v0.9.24 (blastp mode with “-k 1” parameter) [61] against RefSeq non-redundant protein database [62]. Any protein with the best hit protein annotation containing “Unknown”, “Hypothetical”, “DUF (Domain of unknown function)” or “Uncharacterized” words were regarded as hypothetical protein. Whether the pan-genome is open- or close-pan-genome was determined following [16], in which the exponential regression for new genes was fitted to the power law $n=k{N}^{-\alpha }$. Whether the pan-genome is open or close is determined by $\alpha$, in which the pan-genome is open if $\alpha >1$ and close if $\alpha \le 1$.

### Gene clusters annotation

To annotate gene clusters, we used the sequence from representing genes to query several resources. For antibiotic resistance annotation, Resfam [19] and Comprehensive antibiotic resistance database (CARD) [18] were used. CARD is a manually curated database that represents mostly experimentally validated resistance mechanisms, while Resfam database consists of optimized Hidden Markov model for computational detection of protein families validated for antibiotic resistance [19]. CARD annotations were retrieved from RGI tool v5.1.1 with default parameter. CARD antibiotic resistance ontology (ARO) 3.1.1 were downloaded on February, 2021 to interpret RGI results. Resfam HMM database v1.2 and metadata v1.2 were downloaded from the Resfam website (http://www.dantaslab.org/resfams). For functional annotations of genes, pathway, domain and Gene Ontology (GO) annotations were retrieved from EMBL Interproscan v5.48–83 [52]. Cluster of Orthologous Group (COG) annotation was identified using eggNOG HMM model [63] via HMMER v3.2.1 [64] with E-value cutoff set as 1e-10.

### Pan-genome-wide association study to identify putative resistance genes

To identify gene clusters associated with resistance, we compared presence/absence patterns of the gene clusters against resistant phenotype annotations (provided by and downloaded from PATRIC database [55, 56]) using Scoary [17]. Scoary performs Fisher’s Exact Test to identify variants significantly associated with trait and then incorporates phylogenetic structure to look for the mostly likely causal variant. The Scoary analysis was performed with default parameters. Genes with odds ratio > 8 and false discovery rate (FDR) < 0.05 were defined as putative resistance genes.

### Co-inheritance network

Proteins with relevant functions are more likely to be inherited together throughout the evolutionary process [20]. Previously Shin et al. showed that combining 3 co-inheritance network from 3 domains of life respectively increases precision and coverage [20]. To increase coverage on accessory genes, we trained two co-inheritance networks built against different sets of target genomes: ESKAPE genomes (*Enterococcus faecium*, *Klebsiella pneumonia*, *Acinetobacter baumannii*, *P. aeruginosa*, and *Enterobacter species*, which are groups of Gram-negative pathogen commonly share virulence genes), and all RefSeq prokaryotic genomes (ftp://ftp.ncbi.nlm.nih.gov/genomes/refseq/).

The construction of each co-inheritance network was similar to [20]. Firstly, the amino acid sequences of all representing genes in the *E. coli* pan-genome were extracted and mapped against the protein sequences of target genomes using DIAMOND v0.9.24 (with e-value cutoff 0.001) [61]. The e-values were then normalized by second best e-value of each genome into “hit score” between zero and one by the following equation [20]:

$${\hbox{Hit score}}=\left\{\begin{array}{cc}\frac{-\mathrm{ln}\left(evalue\right)}{-\mathrm{ln}\left(second \;best\; evalue\right)}& 0<evalue<1\\ 1& evalue=0\\ 0& evalue\ge 1 \;or\; no\; hit\end{array}\right.$$

A high hit score signifies that the query gene is more likely to have a similar counterpart present in the target genome. A pair of query genes with similar “hit score profile (a vector of length the number of target genomes, a.k.a. phylogenetic profile)” among all target genomes indicates that they may be co-inherited. Mutual information (I) was then used to estimate the similarity between phylogenetic profiles [20]. All query-target pairs of hit scores are discretized into 200 bins with equal intervals; the hit scores are then ordered in each bin to derive the joint probability of the query-target pair. The entropies $H\left({q}_{i}\right)$, $H\left({q}_{j}\right)$, and the mutual information $I\left({q}_{i},{q}_{j}\right)$ are calculated as

$$H\left({q}_{i}\right)=-\sum p\left({q}_{i}\right)\mathrm{ln}\left(p\left({q}_{i}\right)\right)$$

$$I\left({q}_{i},{q}_{j}\right)=H\left({q}_{i}\right)+H\left({q}_{j}\right)-H({q}_{i},{q}_{j})$$

### Domain-sharing network

Protein domains are essential regions that determine protein functions, and genes with similar function usually share the same protein domains. The co-functional links inferred from protein domains are determined following the use of weighted mutual information (WMI) [21], in which rarer domains are weighted more significantly to account for more specific functions. The detailed steps are described as follows.

Given protein domain matrix $M$ with $m$ protein and $n$ domains, each protein domain $j$ is assigned weight ${w}_{\mathrm{j}}$ as:

$${w}_{j}=\frac{{\sum }_{k=1}^{n}{\sum }_{l=1}^{m}{c}_{kl}}{{\sum }_{k=1}^{n}{c}_{kj}}$$

where *C*_xy_ is the abundance of the *x*_th_ domain for *y*_th_ protein. The weighted mutual information ${I}_{w}$ of two proteins $X$ and $Y$ are calculated from weighted entropy ${H}_{w}\left(X\right)$ as:

$${I}_{w}\left(X, Y\right)= {H}_{w}\left(X\right)+{H}_{w}\left(Y\right)-{H}_{w}\left(X,Y\right)$$

Weighted entropy ${H}_{w}\left(X\right)$ is defined as:

$${H}_{w}\left(X\right)=-\sum_{t\in \left\{{0,1}\right\}}\left\{{p}_{w}\left(X, t\right)\cdot log {p}_{w}\left(X, t\right)\right\}$$

And probability ${p}_{w}\left(X, t\right)$ is assigned as:

$${p}_{w}\left(X, t\right)=\frac{\sum_{j\in \left\{j|c{X}_{ji}=t\right\}}{W}_{j}}{{\sum }_{j=1}^{m}{w}_{j}}$$

Similarly, joint entropy ${H}_{w}\left(X,Y\right)$ is estimated as:

$${H}_{w}\left(X,Y\right)=-\sum_{t\in \left\{\mathrm{00,01,10,11}\right\}}\left\{{p}_{w}\left(XY, {t}_{1}{t}_{2}\right)\cdot log {p}_{w}\left(XY, {t}_{1}{t}_{2}\right)\right\}$$

$${p}_{w}\left(XY, {t}_{1}{t}_{2}\right)=\frac{\sum_{j\in \left\{j|c{X}_{j}={t}_{1}, c{Y}_{j}={t}_{2}\right\}}{W}_{j}}{{\sum }_{j=1}^{m}{w}_{j}}$$

### Protein–protein interaction network

The STRING database was downloaded from [22]. DIAMOND v0.9.24 [61] was employed to identify the *E. coli* representing genes that can be mapped to the STRING database (blastp mode; -id 0.7). The subset of the STRING nodes was then extracted by getting only nodes that can be mapped to the *E. coli* representing genes along with the edges that connected the extracted nodes.

### Benchmarking with GO term similarity score

To benchmark each network, biological process GO term semantic similarity score (GOsim) was calculated using GOSemSim 2.12.0 using the Wang method [23, 24]. Each protein can be associated with multiple GO terms. The score of multiple term pairs between two proteins are aggregated using the BMA (best-match average strategy) method [23, 24]. With gene pair with GOsim > 0.6 labelled as “true interaction”, log likelihood score (LLS) was calculated following [12, 14, 20, 25]. Briefly the interval distribution of each network is estimated for its likelihood of interaction as

$$LLS = ln\left( {\frac{{P(L|E)/P\left( { \sim L/E} \right)}}{{P\left( L \right)/P\left( { \sim L} \right)}}} \right)$$

where *L* indicates the true interaction, and *E* (supporting evidence) corresponds to network score intervals. A high LLS score can be interpreted as having a greater chance of having highly similar GO terms.

Positive predictive ratio (PPV) was calculated to quantify the percentage of finding a “true interaction” in network as

$$PPV=\frac{\#edges \;labelld \;as \;true \;interaction}{\# \;edge \;with \;score \;higher \;than \;threshold}$$

And coverage was calculated to estimate how many genes (nodes) of interest can be detected using the network.

$$coverage=\frac{\# \;nodes\; detected \;in \;network}{\# \;input \;nodes}$$

By plotting the PPV-coverage tradeoff with different network scoring thresholds, the area under curve was determined to estimate how accurate and complete the network cover interactions of the genes of interest.

### Integrating distinct networks using random forest regressor

To infer GOsim for gene pairs, we trained a random forest regressor using Scikit-learn 0.22.2 [65] with network scores from EskapeNet, RefseqNet, DomainNet and StringNet. For EskapeNet, RefseqNet and DomainNet, we found that edges lower than 70th percentile consist of LLS = 0, indicating the absence of information for predicting GOsim scores and were removed from training. StringNet only contains high scoring edges and hence was not filtered at all. All missing scores were filled with − 1. To avoid overfitting, the training was conducted on 75% of the data while the rest 25% of the data was retained in the test set for evaluation purpose. The integrated network, dubbed PangenomeNet with random forest predicted score was used for downstream analysis.

### Network visualization

Network visualization was conducted using Cytoscape v3.7.1. Network statistics were calculated using Python 3.6.7 and networkx 2.2 [66].

### Data-driven ontology

Pairwise scores from the PangenomeNet was fed into data-driven ontology (DDOT) CliXO algorithm [48, 67], which facilitates inference, visualization and alignment of biological hierarchies. After the ontology was inferred, terms were aligned to Gene Ontology (GO) [49] and Antibiotic Resistant Ontology (ARO) [18] with false discovery rate (FDR) < 0.05. Terms were assigned to either GO or ARO terms, depending on which term shares a higher similarity in gene composition. Terms without existing ontological term alignment were curated manually by inspecting their protein annotation based on non-redundant (NR) protein database [62].

## Supplementary Information

**Additional file 1.** This DOCX document contains supplementary notes and supplementary figures S1 to S8. **Fig. S1.** Pan-genome growth curve fitting. **Fig. S2.** Hypothetical genes involve in multiple antibiotic resistance. **Fig. S3.** GO enrichment analysis of meropenem resistance associated genes. **Fig. S4.** GO term similarity score distribution of (A) all gene pairs; (B) cumulative distribution of core-core and accessory-accessory edges. **Fig. S5.** The distribution of individual networks (including STRING, RefSeq, ESKAPE, and Domain-sharing networks) associated with log-likelihood scores (LLS; with GO term similarity > 0.6). **Fig. S6.** (A) Edge and (B) node contribution of each network (C) Unique nodes are all accessory genes. **Fig. S7.** Power-law distribution and component size of all networks. **Fig. S8.** Genome statistics of the excluded and included genomes.

**Additional file 2: Table S1.** Genome ID and statistics for Escherichia coli genome downloaded from PATRIC on February 2021.

**Additional file 3: Table S2.** This compressed csv file contains gene cluster annotation of the pan-genome.

**Additional file 4: Table S3.** Scoary-detected resistant gene annotation statistics.

**Additional file 5: Table S4.** GO enrichment results for all Scoary-detected resistant genes.

**Additional file 6: Table S5.** Resistant gene subnetwork scores. Node annotations are in Table S6.

**Additional file 7: Table S6.** This compressed csv file contains all resistant gene annotation.

**Additional file 8: Table S7.** Term enrichment result for the pan-resistome ontology.

**Additional file 9: Table S8.** Pan-genome GO term consistency under different amino acid cutoff.

**Additional file 10: Table S9.** This compressed csv file contains presence absence pattern of all genes in the pan-genome across all E.coli species included in this paper.

**Additional file 11: File S10.** FASTA file for representing gene of all pan-genome.

## Data Availability

The datasets (genomes and AMR phenotypes) are available at PATRIC database (https://www.patricbrc.org/). Pan-genome annotations, presence-absence pattern and networks are available as Additional file 3: Table S2 and Additional file 10: Table S9. The representing gene sequences of each of the gene clusters are available at Additional file 11: Table S10. Network and ontology visualizations are available at Network data exchange (NDex) (http://www.ndexbio.org/#/networkset/67b112d7-a23b-11eb-9e72-0ac135e8bacf). The source code used to construct each subnetworks are as follows: Domain-sharing network code is available at https://github.com/algaebrown/weighted_mutual. Co-inheritance network code is available https://github.com/algaebrown/co-inheritance. Other implementation code is available at https://github.com/algaebrown/resistanceExp.

## Abbreviations

AMR: Antimicrobial resistance
MRSA: Methicillin-resistant *Staphylococcus aureus*
LLS: Log-likelihood score
GO: Gene ontology
ARO: Antibiotic resistant ontology
