# Supplementary material for: PangenomeNet: a pan-genome-based network reveals functional modules on antimicrobial resistome for Escherichia coli strains
Source: BMC Bioinformatics. 2021 Nov 10;22:548. doi: 10.1186/s12859-021-04459-z (PMC8579557; doi:10.1186/s12859-021-04459-z)
Supplement: Supplementary file 1 — Additional file 1. This DOCX document contains supplementary notes and supplementary figures S1 to S8. Fig. S1. Pan-genome growth curve fitting. Fig. S2. Hypothetical genes involve in multiple antibiotic resistance. Fig. S3. GO enrichment analysis of meropenem resistance associated genes. Fig. S4. GO term similarity score distribution of (A) all gene pairs; (B) cumulative distribution of core-core and accessory-accessory edges. Fig. S5. The distribution of individual networks (including STRING, RefSeq, ESKAPE, and Domain-sharing networks) associated with log-likelihood scores (LLS; with GO term similarity > 0.6). Fig. S6. (A) Edge and (B) node contribution of each network (C) Unique nodes are all accessory genes. Fig. S7. Power-law distribution and component size of all networks. Fig. S8. Genome statistics of the excluded and included genomes. [file 12859_2021_4459_MOESM1_ESM.pdf]

PangenomeNet: A Pan-genome-based Network Reveals Functional Modules on Antimicrobial Resistome for *Escherichia coli* Strains

Hsuan-Lin Her, Po-Ting Lin, and Yu-Wei Wu

Supplementary Materials

Supplementary Figures S1-S8
Supplementary Table S1-S10

All resistome network is available on Ndex (Network data exchange):
http://www.ndexbio.org/#/networkset/67b112d7-a23b-11eb-9e72-0ac135e8bacf?accesskey=954cbaa3fd709f9991a8e06248c939a5a391c7ad9cc72b675d5cefe3c175bfc1

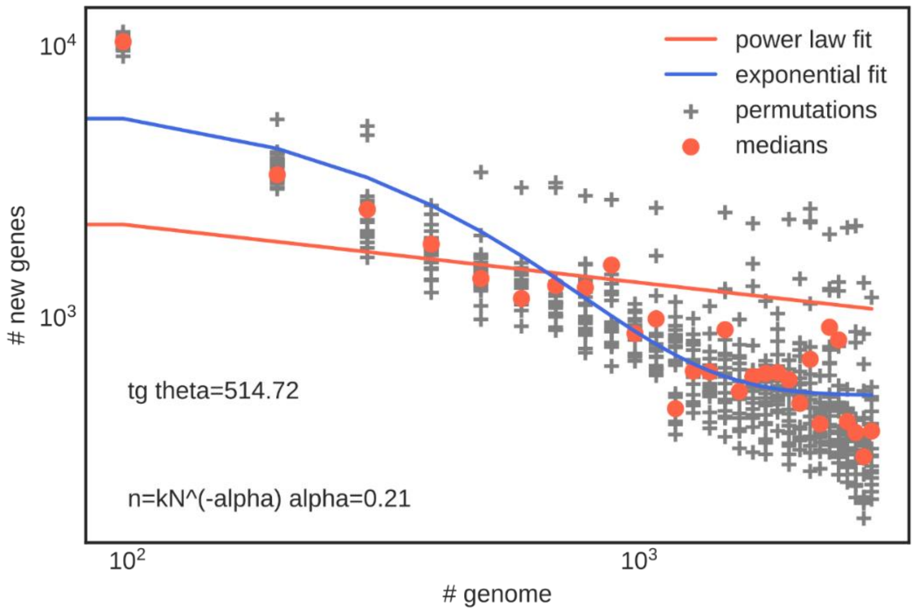

Supplementary Figure S1. Pan-genome growth curve fitting.

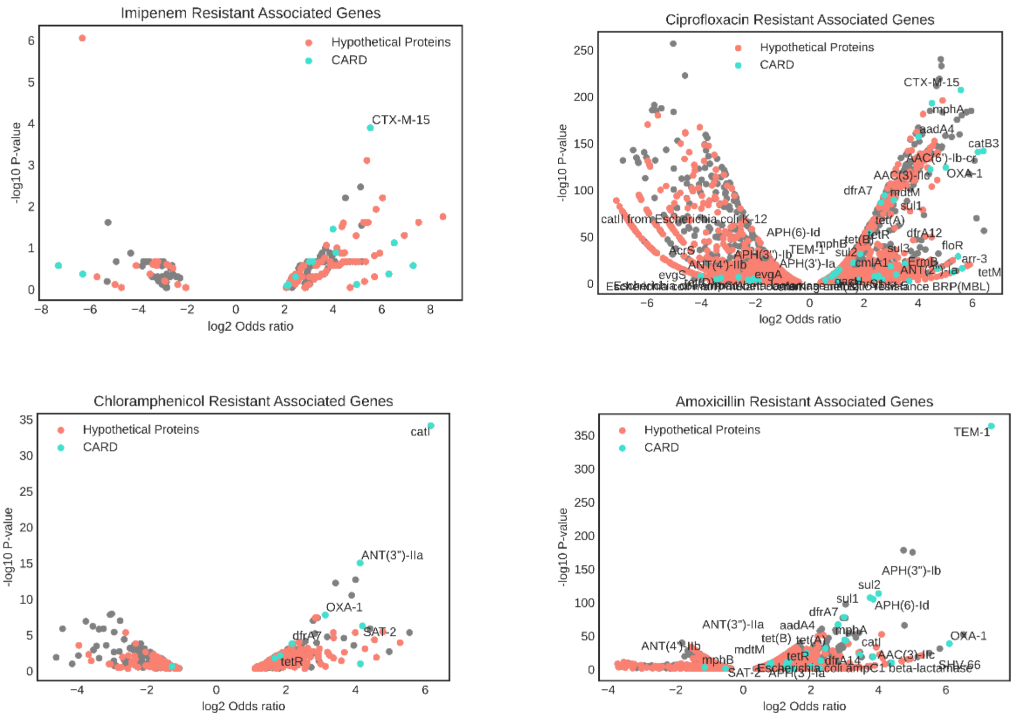

Supplementary Figure S2. Hypothetical genes involve in multiple antibiotic resistance.

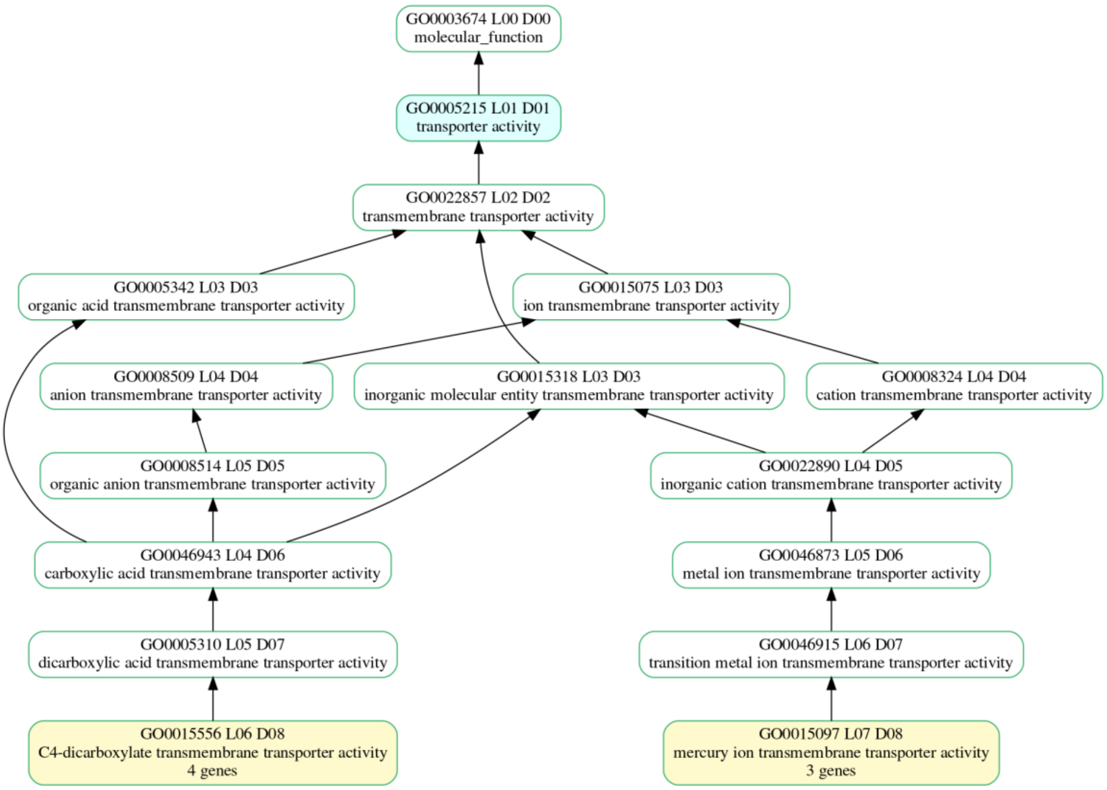

Supplementary Figure S3. GO enrichment analysis of meropenem resistance associated genes.

(A)

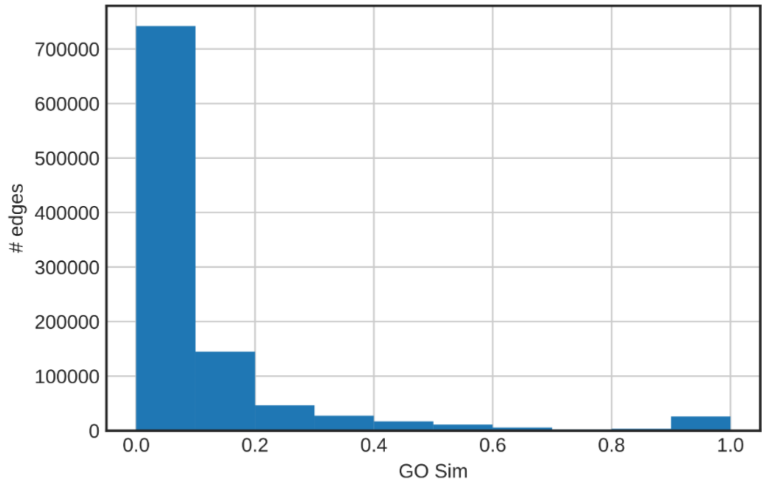

(B)

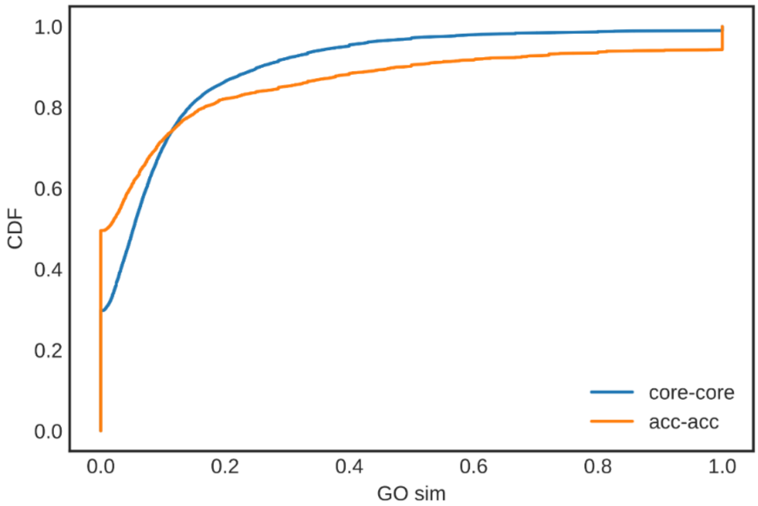

Supplementary Figure S4. GO term similarity score distribution of (A) all gene pairs; (B) cumulative distribution of core-core and accessory-accessory edges.

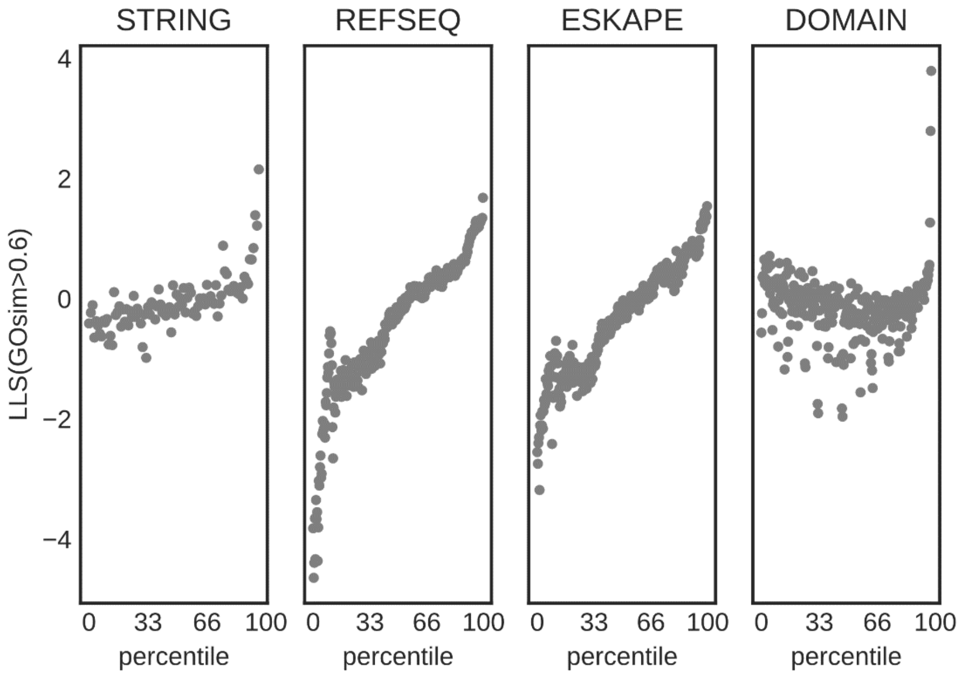

Supplementary Figure S5. The distribution of individual networks (including STRING, RefSeq, ESKAPE, and Domain-sharing networks) associated with log-likelihood scores (LLS; with GO term similarity > 0.6).

(A)

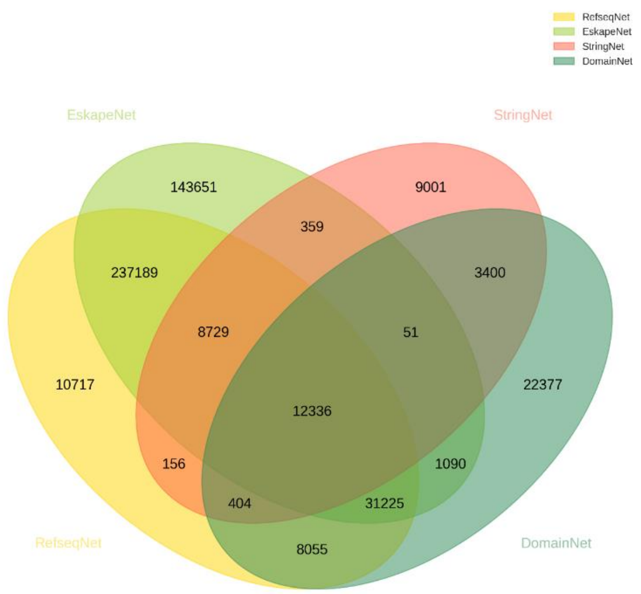

(B)

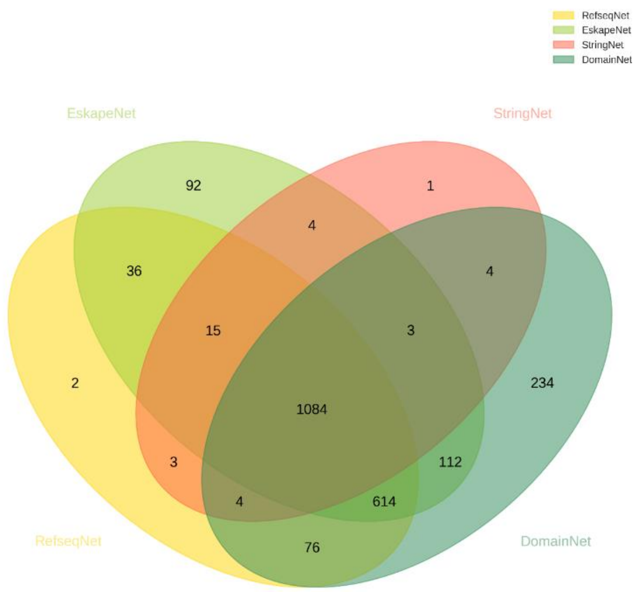

(C)

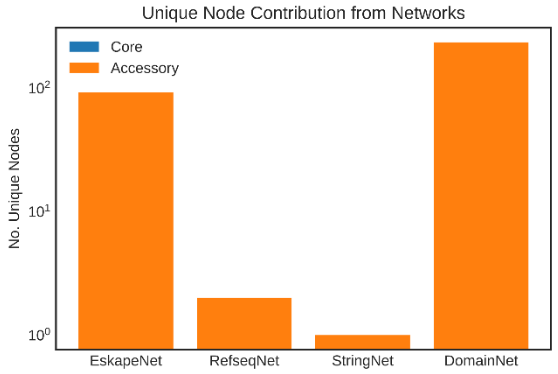

Supplementary Figure S6. (A) Edge and (B) node contribution of each network (C) Unique nodes are all accessory genes.

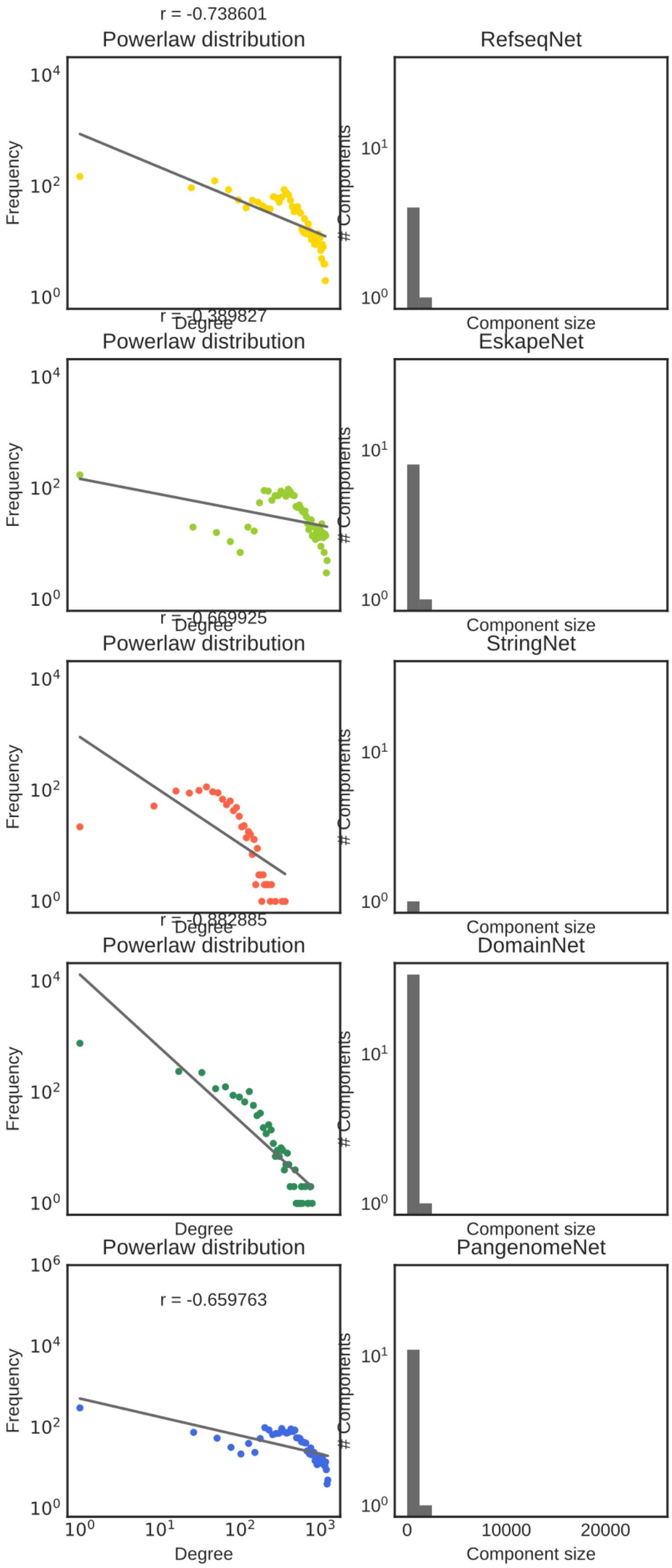

Figure S7 Power-law distributions and component sizes of all networks.

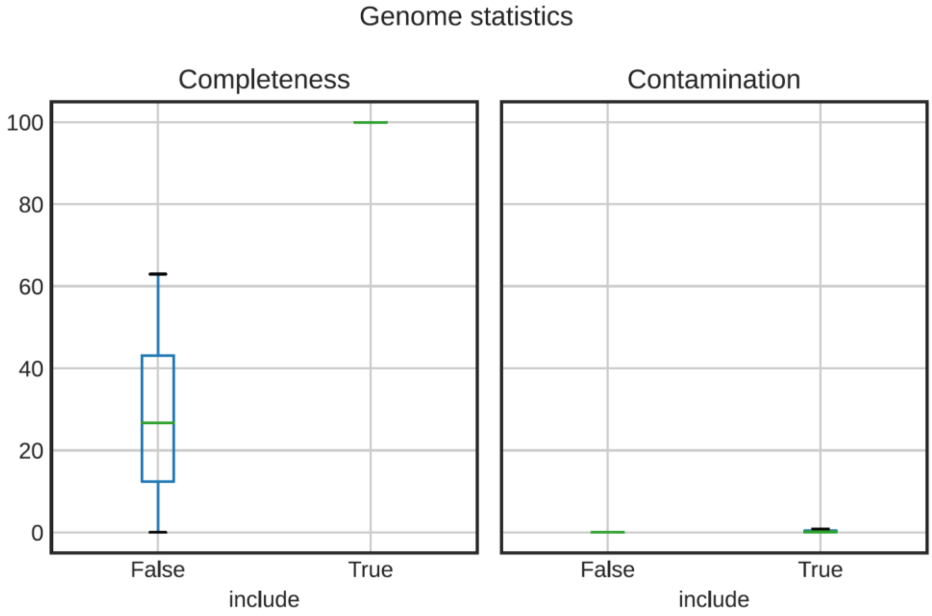

Figure S8 Genome statistics of the excluded and included genomes.

Supplementary Tables

Table S1 Genome ID and statistics for *Escherichia coli* genome downloaded from PATRIC on February 2021

Table S2 Gene cluster annotation of the pan-genome

Table S3 Scoary-detected resistant gene annotation statistics

Table S4 GO enrichment results for all Scoary-detected resistant genes

Table S5 Resistant gene subnetwork scores. Node annotation are in Table S6

Table S6 All resistant gene annotation.

Table S7 Term enrichment result for the pan-resistome ontology

Table S8 Pan-genome GO term consistency under different amino acid cutoff

Table S9 Presence Absence pattern of all genes in the pan-genome across all *E.coli* species included in this paper.

File S10 FASTA file for representing gene of all pan-genome

All Tables are available in .csv/excel format on figshare https://figshare.com/articles/dataset/Supplementary_tables_for_PangenomeNet/14460252.
